# Medical News

**Published:** 1854-10

**Authors:** 


### THE MEDICAL EXAMINER.

PHILADELPHIA, OCTOBER, 1854.

### MEDICAL NEWS.

OPENING OF THE BLOCKLEY HOSPITAL FOR CLINICAL INSTRUCTION. At a Meeting of the Guardians of the Poor, held September 5th, to consider and adopt rules for the government of clinical lectures at the Blockley Hospital, and of other business relating to the subject of clinical instruction, the following report was made and unanimously adopted:

The Clinical Officers shall consist of three surgeons and lecturers on clinical surgery, of which the chief resident physician shall be one, and three physicians and lecturers on clinical medicine, of which the chief resident physician shall also be one, who together shall constitute the Medical Board of the Hospital.

The Medical Board shall arrange the period of the service during the lecture term, in such a manner as will be most conducive to the interests of the Hospital.

The Consulting Surgeons shall always be present at every capital operation, and no operation shall be performed unless advised by at least one Consulting Surgeon and the Chief Resident Physician; the consent of the patient thereto being first obtained.

The Consulting Physicians shall always participate in the recommendation to the Board of Guardians of any measures connected with the medical service, provided the measure has been first recommended by the Chief Resident Physician.

The charge of the Hospital and of the Clinical wards shall be under the Chief Resident Physician, who shall see that such treatment as is directed in consultation is faithfully conducted.

The clinical nurses shall be under the supervision of the Chief Resident Physician, in regard to all ward duties.

No patient shall be transferred from the Hospital to the Clinical Wards except by the order of the Chief Resident Physician, and no patient shall be discharged from the Clinical Wards except with the consent of the consulting officers.

All diversity of opinion in relation to the duties of the medical officers, whether consulting or resident, shall be referred to the Hospital Committee.

Drs. Henry H. Smith and D. H. Agnew were appointed the Consulting Surgeons; and Drs. John L. Ludlow and R. T. Coleman the Consulting Physicians.

On the resignation of Dr. Coleman, at a subsequent meeting, Dr. Caspar Morris was elected in his place.

---

We have received the second annual announcement of the Polytechnic College of the State of Pennsylvania. This excellent school of instruction supplies a want much needed in our country. Courses of lectures are delivered on the different branches, and pupils are practically exercised and made acquainted with them. Every facility is, in fact, afforded to students desirous of becoming acquainted with Mining, Engineering, and the higher mechanical and agricultural arts. So meritorious an institution deserves to be successful.

---

Dr. Walter Channing has resigned the professorship of Midwifery and Medical Jurisprudence, in the Medical School of Harvard University. Dr. D. Humphreys Storer has been appointed to the vacant chair.

---

Professor Bartlett, of the College of Physicians and Surgeons of New York, will not be able to lecture during the coming session, on account of ill-health. Dr. J. M. Smith will deliver the course on Materia Medica. Prof. Clark will lecture on Practice, as well as Pathology. Dr. Dalton will give the course on Physiology.

---

Dr. Sanford B. Hunt, editor of one of our most esteemed exchanges, the Buffalo *Medical Journal*, has been appointed to the chair of Anatomy, in the University of Buffalo, in the place of Dr. E. M. Moore, resigned.

The corner stone of a female medical college, to cost $125,000, has been laid at Richmond, Va.—*Boston Med. and Surg. Jour.*

Obituary.—John A. Swett, M. D., Professor of the Practice of Medicine, &c., in the University of New York, and well known by his work on the Diseases of the Chest, is, we regret to see by the papers, deceased.

We regret to announce the death of Dr. John P. Hiester, of Reading, aged 52 years. Dr. Hiester was the late President of our State Convention.

Erratum.—In our last number, Dr. Harting was quoted as having used quinidine in ague. It should have been written *quinoidine*—another substance.

Omission.—Under the title of the first article of the present number, should have been written the words, "As read before the College of Physicians."

